# Performance-Based Financing to Strengthen the Health System in Benin: Challenging the Mainstream Approach

**DOI:** 10.15171/ijhpm.2017.42

**Published:** 2017-04-15

**Authors:** Elisabeth Paul, Mohamed Lamine Dramé, Jean-Pierre Kashala, Armand Ekambi Ndema, Marcel Kounnou, Julien Codjovi Aïssan, Karel Gyselinck

**Affiliations:** ^1^ Economie politique et économie de la santé, Faculté des Sciences sociales, Université de Liège, Liège, Belgium.; ^2^ PASS-Sourou Programme, Belgian Development Agency, Benin.; ^3^ Comé District, Ministry of Health, Comé, Benin.; ^4^ Atacora-Donga Departmental Health Team, Ministry of Health, Natitingou, Benin.; ^5^ Belgian Development Agency, Brussels, Belgium.

**Keywords:** Performance-Based Financing (PBF), Health System Strengthening (HSS), Local Health System, Benin, Low and Middle-Income Countries (LMICs), Demand-Side Actors

## Abstract

**Background:** Performance-based financing (PBF) is often proposed as a way to improve health system performance. In Benin, PBF was launched in 2012 through a World Bank-supported project. The Belgian Development Agency (BTC) followed suit through a health system strengthening (HSS) project. This paper analyses and draws lessons from the experience of BTC-supported PBF alternative approach – especially with regards to institutional aspects, the role of demand-side actors, ownership, and cost-effectiveness – and explores the mechanisms at stake so as to better understand how the "PBF package" functions and produces effects

**Methods:** An exploratory, theory-driven evaluation approach was adopted. Causal mechanisms through which PBF is hypothesised to impact on results were singled out and explored. This paper stems from the co-authors’ capitalisation of experiences; mixed methods were used to collect, triangulate and analyse information. Results are structured along Witter et al framework.

**Results:** Influence of context is strong over PBF in Benin; the policy is donor-driven. BTC did not adopt the World Bank’s mainstream PBF model, but developed an alternative approach in line with its HSS support programme, which is grounded on existing domestic institutions. The main features of this approach are described (decentralised governance, peer review verification, counter-verification entrusted to health service users’ platforms), as well as its adaptive process. PBF has contributed to strengthen various aspects of the health system and led to modest progress in utilisation of health services, but noticeable improvements in healthcare quality. Three mechanisms explaining observed outcomes within the context are described: comprehensive HSS at district level; acting on health workers’ motivation through a complex package of incentives; and increased accountability by reinforcing dialogue with demand-side actors. Cost-effectiveness and sustainability issues are also discussed.

**Conclusion:** BTC’s alternative PBF approach is both promising in terms of effects, ownership and sustainability, and less resource consuming. This experience testifies that PBF is not a uniform or rigid model, and opens the policy ground for recipient governments to put their own emphasis and priorities and design ad hoc models adapted to their context specificities. However, integrating PBF within the normal functioning of local health systems, in line with other reforms, is a big challenge.

## Background

Health systems in many low- and middle-income countries (LMICs) face a number of structural problems and inefficiencies. In view of the lack of success of most reform attempts, performance-based financing (PBF) has been proposed as a way to catalyse comprehensive reforms and help improve health system performance.^1^ PBF is a supply-side form of results-based financing whose core features are the following: performance-based incentives are earned by service providers; payments are targeted at individual health facilities and administrations, often with trickle-down effect to health workers; there is usually some split of functions between regulation, purchasing, fund-holding, verification and service delivery; payments are linked to outputs, modified by quality indicators.^2^ Following a few positive experiences, especially in Rwanda,^3,4^ PBF has fastly expanded in sub-Saharan Africa in the past decade. To our knowledge, PBF implementation in LMICs has almost always been supported by donors, particularly the World Bank which administers the Health Results Innovation Trust Fund (HRITF) created in 2007 to support results-based financing approaches in the health sector (see https://www.rbfhealth.org). The Belgian Development Agency (BTC) has also supported PBF in several countries among which Rwanda and Burundi.

In Benin, after two inconclusive experiences led by the Ministry of Health (MoH) in 2007 and BTC in 2008-2009, PBF was again experimented in 2012 through a World Bank-supported pilot project in eight health districts. The MoH then requested BTC to follow suit in the five districts it was supporting through a health system strengthening (HSS) project: Comé-Bopa-Grand Popo-Houeyogbé (CBGH), Klouèkanmé-Toviklin-Lalo (KTL) and Aplahoué-Djakotomey-Dogbo (ADD) in the Mono-Couffo region (South Benin); Bassila, and Djougou-Ouaké-Copargo (DOC) in the Atacora-Donga region (North Benin). In 2015, PBF was scaled up in all districts of the country thanks to financial support from Gavi and the Global Fund to Fight Aids, Tuberculosis and Malaria (GFATM) which adopted the World Bank’s approach. The latter is similar to that implemented in other countries (Rwanda, Burundi) and relies on a project coordination unit for piloting, on an external firm for verification of results, and on community-based organisations for counter-verification. However, as explained below, BTC has developed a promising alternative, more integrated and less resource-consuming approach which is grounded on existing domestic institutions and networks, aims to strengthen the local health system, and uses peer review for verification of results and health service users’ platforms for counter-verification. Since 2015, the MoH has initiated a joint process aimed at harmonising the PBF approaches in view of rendering it country-led and sustainable.

As pointed by a recent literature survey, what PBF actually entails is not straightforward, and existing PBF schemes around the world differ on almost every single of its usual composing elements; moreover, the exact mechanisms through which PBF financial incentives, contractual features and ancillary components such as increased control and accountability mechanisms operate are not well understood.^5,6^ Therefore, the objective of this paper is to analyse and draw lessons from the experience of the BTC-supported PBF alternative approach developed in Benin, taking account of its context, and to explore the mechanisms at stake so as to better understand how the “PBF package” functions and produces its effects. This is important to demonstrate that PBF should not be viewed as a uniform or rigid model and on the contrary, governments should adapt PBF designs to their context specificities and priorities. The institutional aspects, the role of demand side actors, the issues of ownership and cost-effectiveness are given particular attention. The paper concludes by discussing sustainability issues in relation to PBF.

## Methods

Since it aims to “open the black box” of PBF,^5^ this paper adopted an exploratory, theory-driven evaluation approach.^7^ Indeed, the successive BTC health sector support programmes have been designed as pilot, research-action programmes aimed at testing a number of hypotheses supposed to contribute to increasing health system performance through strengthening the health system at central, intermediate (departmental) and local (district) levels. The technical and financial document orienting the current programme, called PASS-Sourou, lays out a complex intervention logic explaining how planned activities are hypothesised to interact towards five expected results, which together are hypothesised to strengthen the health system’s three key dimensions: supply-side, demand-side and governance. Noticeably, PBF is not isolated as a single activity or result, but is referred to as a transversal approach contributing to several results, with activities relating both to the supply-side and demand-side of the system. The programme orientation document also specifies that the PBF model should keep on being tested so as to ensure it is people-centred, equity-oriented and sustainable.^8^

**Figure 1:**
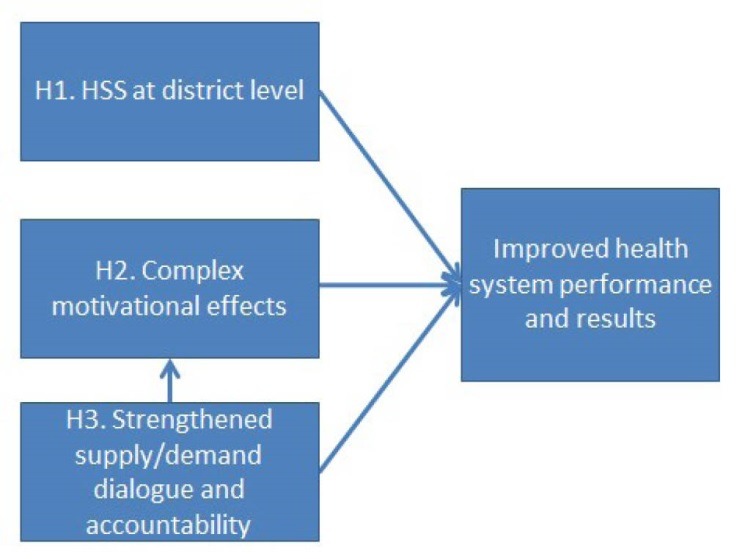
Hypotheses on How the BTC Alternative PBF Model Impacts on Results. Abbreviations: HSS, health system strengthening; BTC, Belgian Development Agency; PBF, performance-based financing.

From this programme orientation document as well as from authors’ participation in the design and development of the BTC alternative PBF approach since 2012, and through an iterative process, three causal mechanisms through which PBF is hypothesised to impact on results were singled out, that we intend to explore in this paper: (*i*) comprehensive HSS at district level; (*ii*) acting on health workers’ motivation through a complex package of incentives; and (*iii*) increased accountability by reinforcing dialogue with demand-side actors. This set of hypotheses may basically be outlined as shown in Figure 1.

Since the BTC alternative PBF approach has been conceived as a systemic intervention with a possibly broad range of effects on the health system, and thus since we intend to analyse the interactions between PBF and the local health system, we broadly followed the PBF monitoring & evaluation framework proposed by Witter and colleagues.^2^ Therefore results are presented based on analyses of: (1) the influence of context over PBF; (2) policy formulation; (3) design; (4) implementation; (5) effects of PBF on health systems.

This paper stems from the co-authors’ capitalisation of experiences, that is usually defined as transformation of experience into shareable knowledge.^9^ It is issued after 4 years of research-action, at a time when, on the one hand, both World Bank and BTC funds available to support PBF are ending (respectively in June and September 2017); and on the other hand, the MoH is urged to take stock of donor-supported PBF schemes to design its own model and find budget rooms to finance it. The theory-driven evaluation approach adopted was conceived through an iterative process and relies on crossed perspectives from all the co-authors. They have all been involved either directly or indirectly in the BTC programme – be it as coordinator of the overall HSS programme, technical assistant, MoH recipient at departmental or district level, headquarter backstopping or consultant; one of them is also an academic with extensive experience in Benin. For each of the five domains analysed below, mixed methods were used to collect, triangulate and analyse information, including: financial and technical data produced by the BTC-supported project over four years (third quarter 2012 to third quarter 2016), routine data about the health system and results, existing records and studies on the PBF approaches implemented in Benin; key stakeholders interviews performed at national and operational levels during a previous research^10^; and mainly participative observation of PBF implementation – including on the field by those co-authors involved at departmental and district levels, and also during the PBF harmonisation process launched by the MoH in 2015 (as members of the four joint missions and other technical workshops), which enabled to compare the pros and cons of the two PBF models implemented to date.

## Results

### Influence of Context over Performance-Based Financing

As argued by Witter and colleagues, since health systems are complex adaptive systems, it is necessary to include the context in understanding and documenting PBF, and to monitor the continuous interactions between the context and other PBF domains (or features) over time.^2^ Benin makes no exception and PBF was introduced in a complex system plagued with important bottlenecks. The National Health Forum organised in November 2007, which gathered some 600 participants, performed an in-depth assessment of the health sector context, and formulated a number of recommendations which guided the design in 2009 of the new 10-year health sector development plan. Main issues identified relate to governance in the health sector (poor performance, lack of leadership), poor quality of healthcare, inadequate infrastructure and equipment, and the need to valorise human resources for health – some of them will later be targeted by PBF (governance, quality, human resources). However, if the importance of establishing a management system based on a culture of performance, accountability and results was referred to, the possibility of introducing PBF was not mentioned. Rather, “orthodox” solutions to improve governance and staff motivation (such as salary increases and distance allowances) were proposed. An emerging priority was to install a consistent quality insurance system in the health sector.^11^ As for the 2009-2018 Health Sector Plan, it also only vaguely refers to the need to design mechanisms enabling to incentivize staff retention and performance, through improving working conditions, valorisation of performance, and adoption of retention measures. This plan does not mention PBF.^12^

Introduction of PBF in the health sector in Benin was obviously propelled by its international popularity, which also triggered additional donors later during the implementation phase. As explained below, PBF was introduced with support from various donors – mainly the World Bank, but also BTC who piloted a performance premium scheme in one district in 2008 and 2009, plus other donors who also initiated punctual PBF schemes (notably UNICEF at community level). Another influential contextual element deals with the creation in 2011 of a joint HSS platform, following the signature in November 2010 of the first “Compact” between the MoH and five donors within the framework of the International Health Partnership and related initiatives. The HSS platform gathers the World Bank, GFATM, Gavi and BTC around the MoH, with support from the World Health Organization (WHO). In this context, the four main donors agreed on a geographical distribution of their HSS support so as to cover all the 34 health districts of the country: 8 were already supported by the World Bank and 5 by BTC; Gavi and the GFATM agreed to support respectively the 2 and 19 remaining districts. Involvement of new donors will prompt a harmonisation process and thus adaptation of some elements of the design of both PBF approaches.

### Policy Formulation

The World Bank project’s Appraisal document candidly explains that:

“In 2007, Benin started to test [Results-Based Financing (RBF)] in 3 districts. According to an evaluation carried out in 2008, the experiment was plagued with numerous issues related to implementation […]. After several workshops and study tours (in Rwanda), the MoH decided to continue and even to scale-up this experience, but only after a deep redesign of the RBF mechanism. The MoH then applied for a RBF grant ($11 million) under the Multi-Donor Trust Fund for Health Results Innovation […]. [H]ealth workers’ unions were involved very early in the policy dialogue, as they were initially quite reluctant to RBF. Again, after several workshops and one study tour to Rwanda, they became strongly supportive of the mechanism. Finally, in August 2009, the Cabinet (“*Conseil des Ministres*”) officially declared RBF a key priority for the health sector.”^13^

The World Bank approach is a mainstream one. It is very much inspired by the Rwandan experience and follows the standards of the World Bank PBF Toolkit.^14^ The project was initially conceived under a randomised control trial enabling to test the efficacy of various PBF designs with a control group. In 2012, following implementation of PBF in the 8 World Bank-supported districts, the MoH requested BTC to also introduce a PBF component in its existing HSS programme. However, BTC developed an alternative approach, more suited to its ongoing intervention. Benin later submitted HSS proposals to Gavi and the GFATM comprising a PBF component based on the World Bank approach, which was presented as the national approach. Since 2015, PBF has been a national strategy however exclusively financed by donors. The World Bank’s project coordination unit coordinates PBF activities in 29 districts, while BTC supports coordination mechanisms in its 2 regions of intervention.

According to our field observations and interviews performed since 2006, PBF is not a home-grown policy but is donor-driven in Benin. Some former MoH top executives were initially opposed to PBF, and it is only after intensive lobbying from the World Bank (including workshops and study tours in Rwanda) that they finally bought the idea. However, ownership of PBF within and outside the MoH remains limited to a few people (see below) – and for long, only the World Bank approach had some kind of visibility outside the project coordination unit and a few MoH top managers. Outside an independent study performed in 2013 and published in 2014,^10^ it is only during the 2015 joint annual health sector review, and later an inter-agency field mission organised in June 2015, that the coexistence of two PBF approaches was discussed by the MoH and its main partners. Consequently, a process aimed at harmonising PBF in view of its sustainability was initiated by the MoH and supported by BTC and other donors. Four joint missions were organised between July 2015 and October 2016. They enabled to identify strengths and weaknesses of each approach in view of refining a Beninese PBF approach, and to inform a wider range of stakeholders about PBF. However, national ownership of PBF is weak, as testified by the fact that a number of key stakeholders within and outside the MoH were only informed about PBF during the second and third joint missions on PBF harmonisation in October 2015 and July 2016.^15,16^ Moreover, in early 2017, there is still no national MoH entity formally in charge of PBF, and no domestic budget to finance it.

### Design

BTC did not adopt the World Bank standard PBF design, which relies on a project coordination unit for overall piloting, strategic purchasing and payments; a purposely-created external verification agency, managed by an international consultancy firm, charged with verification, coaching and technical assistance; and punctually hired community-based organisations for community counter-verification (however, this happened only in 2014). For coherence-sake with focus of its initial HSS support programme as well as for budget constraints, BTC developed an alternative ad hoc approach through an action-research process, with strong involvement of recipients and backing from a research institution charged with scientific support to the programme.^17^ This approach builds on existing institutions and local networks with light external support, and uses peer review for verification. This makes it both more integrated and owned at national and local levels, and less resource consuming. Its main features are described below.

### Performance-Based Financing Coordination

An important feature of PBF in general is separation between the various functions of regulation, financing, purchase of services, service provision and data verification, thus creating a clear division of labour between each player and contributing to transparency.^14^ The BTC-supported alternative approach has entrusted PBF coordination to a steering committee organised quarterly at departmental (provincial) level, consisting of representatives from the departmental health office, donors, mayors, civil society organizations (CSOs), health services users’ platforms (see below), the mutual health organisations’ medical officer, and service providers. This committee is in charge of adapting the overall PBF approach to the local context, deciding on the level of PBF subsidies based on results checked through verification and counter-verification, and managing complaints.

### Purchasing and Payment Functions

The PBF purchasing function is devoted to city councils that take decisions and sign the PBF contracts. The latter are also co-signed by the departmental directorate for health, BTC, steering committee chairman, and each health facility. To date, PBF subsidy payment is still managed by the BTC programme, but stakeholders are now exploring how to delegate it to a national institution.

### Verification of Results

Verification of results reported by health facilities is based on peer review through mixed team supplemented by external stakeholders in order to guarantee independence. At health centre (HC) level, a mixed peer review team composed of a doctor, a midwife, a financial officer, and a nurse coming mainly from the departmental office and an external district leads verification quarterly. It ensures simultaneously verification of quantities along a matrix of quantitative indicators, and quality assessment along another matrix of quality measures. It also draws the sample to be used for counter-verification. At district hospital (DH) level, a mixed peer review team coordinated by the departmental health office and supervised by the mutual health organisations’ medical officer leads verification quarterly. It only controls quality of healthcare since BTC has so far chosen not to include quantitative indicators at hospital level. At the level of the district health management team (DHMT), quantitative indicators are quarterly pre-validated by the local departmental directorate for health, and then audited by another departmental directorate for health and mutual health organisations’ medical officer.

### Counter-Verification

Interestingly – this is to our knowledge the only example in Africa – counter-verification of results at community level is entrusted to health services users’ platforms, which are networks gathering a wide range of relevant stakeholders (representatives from local councils, CSOs/ non-governmental organizations (NGOs), mutual health organisations, community health workers) whose creation and functioning has been supported by BTC for several years. They are both in charge of controlling the effectiveness of care (based on a sampling of HCs records) and conducting satisfaction surveys among HCs and DH patients.

### Quantitative and Qualitative Measures

Overall, the BTC approach puts a lot of emphasis on quality of care: at HC level, bonuses calculated based on actual quantitative indicators (number of cases times “prices” associated to each indicator) are weighed by a qualitative index combining assessment of technical quality and customers’ satisfaction. At DH level, only qualitative measures are taken into account. Finally, once PBF subsidies are allocated to each facility, about 30%-40% is kept by the facility to finance functioning and activities, while the rest is distributed to motivate staff (all categories included) according to a flexible multi-criteria allocation grid.

### Implementation

BTC started to implement PBF at DHMT level in the third quarter (Q3) of 2012. Then it was progressively rolled over at HC and DH level in the three districts of a first department (Mono-Couffo) in the first quarter (Q1) of 2013, and then in the 2 districts of the other department (Atacora-Donga) in the second quarter (Q2) of 2013. The initial design was progressively adapted with revision of indicators and qualitative matrices in Q2 2014 and later in Q3 2015. The criteria for calculation of DHMT subsidies also evolved: they were initially based on implementing routine activities, but now are also linked to the attainment of some performance indicators by HCs in their district. The level of PBF subsidies also increased so as to progressively align on the level of those granted by the World Bank, which were initially much higher. Other adaptations include gradual involvement of private HCs and community health workers in PBF, in line with the relevant national policy documents; as well as the introduction of equity bonuses to provide extra incentives to disadvantaged facilities. BTC had initially programmed a 1.5 million EUR envelope to support PBF until the end of 2015, and then to transfer PBF management to the World Bank. However, this was actually not possible and BTC rose another 1 million EUR funding in order to finance PBF until September 2017. Table 1 shows the main evolutions in quantitative indicators and their associated premium or price at HC level.

**Table 1:** Evolutions in Quantitative Indicators and Their Associated Premium at HC Level, 2013-2016

	**Indicator**	**Price Applied in 2013 (XOF)** ^a^	**Price Applied Q1 2014–Q2 2015 (XOF)**	**Price Applied Q3 2015–Q3 2015 (XOF)**
1.	New outpatient consultation	130	150	200
2.	Attended eutocic birth	2000	2500	3000
3.	Emergency delivery reference	2000	2500	3000
4.	Completely vaccinated children	500	600	1500
5.	Pregnant women fully immunized in tetanus toxoid vaccine (TT2-5)	300	400	1200
6.	Pregnant women who received the 2nd dose of sulfadoxine-pyrimethamine	300	400	1200
7.	Family planning acceptors (end of month)	700	800	1000
8.	Screening for tuberculosis Koch Bacillus +	2000	3500	6000
9.	Tuberculosis cases treated and cured	3500	8000	9000
10.	Referred cases arrived at the DH	700	800	900
11.	Acute severe malnutrition cases detected and clinically treated at HC			1000
12.	Acute severe malnutrition cases referred by the HC and arrived at the DH			1000
13.	Acute severe malnutrition cases treated at HC for up to 28 days and declared cured according to criteria			2000
14.	Children born from seropositive mothers whose delivery respected the protocol and whose PCR 1 test was carried on			10 000

Abbreviations: HC, Health Centre; DH, district hospital. Source: Data collected from the BTC HSS “PASS-Sourou” programme. ^a^ 655.957 XOF = 1 Euro.

Influence of context is noticeable in the fact that adaptations in implementation were particularly prompted by the harmonisation process in view of PBF sustainability initiated in 2015. The MoH launched this process following a recommendation from the health sector’s joint annual review. It was much welcome since the GFATM started to support PBF in 19 districts in Q3 2015, and Gavi in the remaining two districts in Q4 2015. A first joint mission aimed at PBF harmonisation was organised by the MoH (with support from consultants hired by BTC) in July 2015, which enabled to carry out a consensual comparative analysis of the two PBF approaches implemented to identify room for mutual improvements. A roadmap for PBF harmonisation in view of its sustainability was elaborated and later regularly updated, and a number of general principles agreed upon. A second joint mission, organised in October 2015, allowed setting the path for the definition of common, consensual indicators and quality measure matrices. Technical officers from the 2 programmes pursued this work until a third mission organised in July 2016. However, a number of issues were not agreed upon at that time – notably the costing of the new indicator matrices, and choice of common mechanisms and institutions for verification and counter-verification. BTC therefore decided to adapt its indicator and quality matrices, but to keep its pricing level.

Perceptions by local stakeholders about PBF are mixed. A qualitative survey performed in November 2013 in 2 health districts – one supported by BTC, another one by the World Bank – showed that after 1 or 2 years of PBF implementation, field actors welcomed PBF – especially additional supervision and training attached to it – and were overall satisfied with PBF. However, they did not show a sense of ownership and viewed it as “another project.” Local stakeholders also accommodated the operational instructions to suit their constraints (eg, adapted the records they were supposed to fill in). A major issue under both approaches was perception of unfairness of PBF. This arose from the fact that until the introduction of equity bonuses, the subvention of all facilities, whatever their initial endowment, were determined based on the same indicators and quality checklist. Thus disadvantaged facilities lacking material, equipment or staff, were automatically penalised, despite the efforts they made. Moreover, BTC started by giving financial incentives to DHMTs while staff at HC level received only low premiums in the first two years of implementation.^10^ However, since then, BTC has regularly adapted its approach to address the problems raised in departmental steering committees. PBF subsidies were raised, qualitative matrices adapted, and equity bonuses introduced. Moreover, contrary to the World Bank’s, the BTC programme entails an infrastructure and equipment facility, which was used to level out HCs in that respect.

### Costs

Main cost items relate to managing the programme (as long as it is a separate entity), generating and verifying performance data. However, in Benin, the management costs of the integrated PBF approach developed in the BTC programme are not very resource consuming. Table 2 shows the annual PBF management costs under the BTC alternative approach as implemented in 2016, covering 5 health districts for an estimated population of 1 759 925 inhabitants.

**Table 2:** Annual PBF Management Costs Under the BTC Approach as Implemented in 2016 (EUR)

**Cost**	**Euros**
Technical assistance^a^	177 488
General management^b^	65 670
Organisation of steering committees (2 departments)	12 196
Verification of results (peer-review)	106 714
HC level	73 176
DH level	12 196
DHMT level	12 196
Departmental directorate for health level	9147
Counter-verification of results by health services users’ platforms (control of effectiveness of care and customer satisfaction surveys)	28 965
HC level	24 392
DH level	4573
Total management costs per year	391 034

Abbreviations: BTC, Belgian Development Agency; PBF, performance-based financing; DHMT, district health management teams; HC, Health centre; DH, district hospital. Source: Data collected from the BTC HSS “PASS-Sourou” programme based on actual costs in 2015 and 2016. ^a^ Based on assumptions on the relative share of time spent by each technical assistant of the programme to PBF, varying from 12.5% (2 mutual health organisations’ medical officers) to 60% (2 counsellors of the demand-side facility). ^b^ Assumption: 10% of the programme’s overheads plus scientific support budget.

This amounts to 0.22 EUR management cost per person per year. Note that it is possible to complement the current verification strategy to reinforce its independence, for instance by adding external controls on a sample of verification mission, a quality control of satisfaction surveys and community feedback activities: this would raise the cost per person per year to 0.24 EUR.

**Figure 2:**
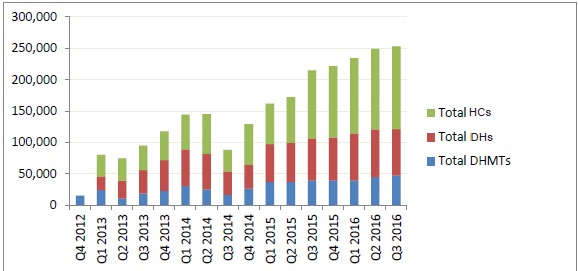
PBF Subsidies Paid by BTC Since Q4 2012^a^.

Abbreviations: DHMTs; district health management teams; PBF, performance-based financing; BTC, Belgian Development Agency; HCs, health centres; DHs, district hospitals.

Source: Data collected from the BTC HSS “PASS-Sourou” programme.

^a^ Reduction in Q3 2014 stems from change in qualitative matrix.

In addition to management costs, one has to add the cost of PBF subsidies. Actual subsidies paid over the period Q4 2012–Q3 2016 are shown in Figure 2.

In 2015, cost of PBF subsidies paid to structures amounted to 0.36 EUR per person per year, to which transaction costs amounting to 0.23 EUR per person per year need to be added, bringing the total PBF cost to 0.59 EUR per person per year. As a matter of comparison, in the eight districts supported by the World Bank project, subsidies paid to DHs amounted to US$0.15 (0.14 EUR) per person per year and subsidies paid to HCs amounted to US$0.95 (0.86 EUR) per person per year in 2015, hence 1 EUR PBF subsidies paid to structures; cost of the external verification agency amounted on average to US$0.15 (0.14 EUR) per person per year for verification, plus US$0.21 (0.19 EUR) per person per year for technical assistance over the period 2012-2015. Note however that this includes neither the general management costs of the project coordination unit, nor counter-verification since it happened only in 2014.^18^

### Effects of Performance-Based Financing on Health Systems

It is difficult to isolate the PBF effects – especially since it does not only comprise financial premiums, but also includes a number of contractual features and “ancillary components” which contribute to its effects.^5^ In Benin as well, PBF *stricto sensu* is complemented by various ancillary supports. It is noticeable that BTC’s alternative PBF scheme is embedded in an HSS programme, hence it was expected to act upon the various building blocks of the health system. A first causal mechanism identified in the process of this theory-driven evaluation is precisely about comprehensive HSS at district level. The HSS programme allows the provision of comprehensive and systemic support to the 5 districts in view of strengthening capacities and developing local health systems. Such comprehensive support comprises integrated supervision by a multidisciplinary team at each level of the health system, from HCs to departmental management teams; continuous in-job training; infrastructure rehabilitation and medical equipment maintenance; reference & counter reference; support to the local governments and representatives from service users. The PBF components are perfectly integrated within the vision of a strong local health system: for instance, PBF verification missions go beyond mere verification of results, but are also opportunities for coaching, retro-information from communities, dissemination of good practices, and progressive development of a quality insurance approach. This coherent approach enables to multiply the benefits from PBF and, according to feedback from DHMTs and healthcare providers, has contributed to improving quality of care. Moreover, consistent local HSS was expressed as a hypothesis to explain that PBF had slightly better results in the districts supported by BTC than those supported by the World Bank (field observation during the fourth joint mission on PBF harmonisation, October 2016).^19,20^

Main observed effects are explained below, as well as other tentative causal mechanisms by which PBF impacts on results.

### Infrastructure and Equipment

The BTC HSS programme comprises a facility aimed at reinforcing infrastructure and equipment, notably to support blood transfusion, emergency neonatal and obstetric care, and hospital hygiene. Moreover, with the introduction of PBF, a share of PBF subsidies is kept at facility level to finance investment on small equipment and activities like motorbikes to facilitate immunisation and mother & chid health outreach activities; repair of ambulances and supervision vehicles; tiling of operating rooms and maternity wards; accommodation for waiting rooms, acquisition of curtains, bed sheets, and hand washing devices contributing to hospital hygiene and patient comfort. Choice of acquisition and/or repair of equipment are oriented in view of improving quality of services according to PBF matrices requirements and standards (technical quality) as well as feedback from community surveys (perceived quality).

### Information Systems

**Figure 3:**
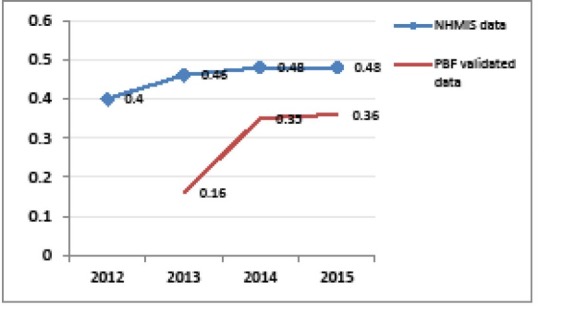
Outpatient Attendance (New Consultations/Person/Year).
Abbreviations: NHMIS, national health management information system; PBF, performance-based financing.

**Figure 4:**
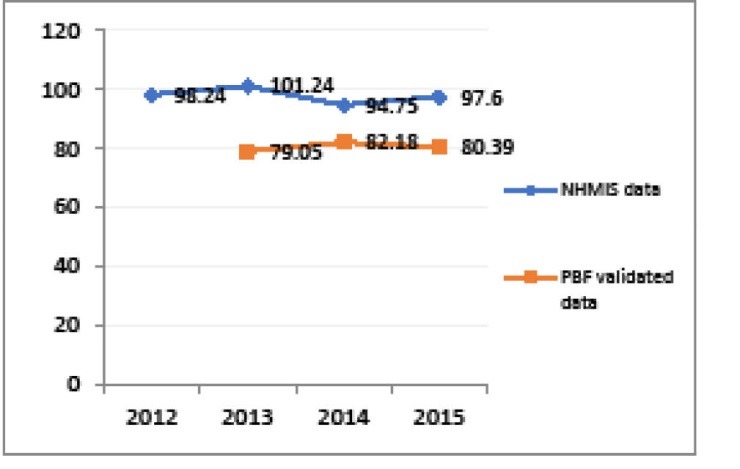
Fully Vaccinated Children (%).
Abbreviations: NHMIS, national health management information system; PBF, performance-based financing.

**Figure 5:**
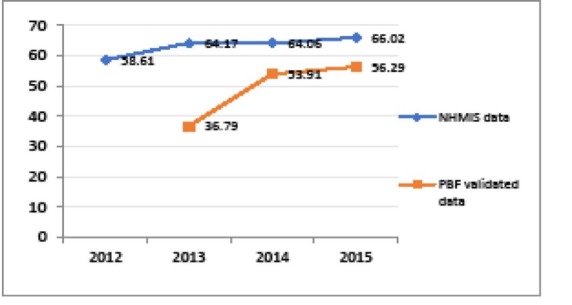
Attended Eutocic Deliveries (%).
Abbreviations: NHMIS, national health management information system; PBF, performance-based financing.

Another building block strengthened in the context of PBF is the national health information and management system (NHIMS). Verification of results enabled to support facilities in better fulfilling the NHIMS. One observes a reduction in the gap between the obviously over-reported NHIMS data and PBF-validated indicators over time in the five districts supported by BTC (see Figures 3-5). Besides, the World Bank project has supported a number of initiatives aimed at improving data management, quality and utilisation and thus synergy between PBF and NHIMS, among which the implementation of the data warehouse district health information system 2 (DHIS2) in all districts in 2015.

### Human Resources

**Figure 6:**
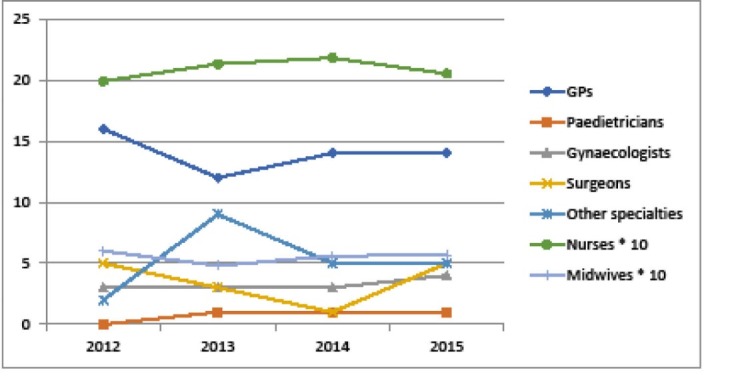
Evolution of Qualified Staff Per Category in the 3 districts Supported by BTC in the Mono-Couffo Department, 2012-2015.
Abbreviations: GPs, general practitioners; BTC, Belgian Development Agency.

Source: HR Department, Mono-Couffo Departmental Health Directorate.

The effect of PBF on human resources is not measured systematically. PBF has not had direct impact on health staff hiring and deployment and overall, the districts supported by BTC did not benefit from major health staff increase, as illustrated in Figure 6. However, PBF had a likely indirect effect on staff mobilisation. For instance, we have observed that some HCs have called for support from neighbouring HCs’ qualified staff to implement some technical activities in order to reach PBF objectives. Two DHs in the Mono-Couffo region took the initiative of entering into partnership with the Faculty of Medicine to host trainees in fourth grade of specialisation in gynaecology and obstetrics. In the context of PBF, some healthcare professionals were hired to fill vital gaps through service contracts (for example, a specialised nurse was hired by a HC in the district of Bassila); however, many did not remain at their assigned position (observation from the BTC “PASS-Sourou” programme). More generally, interviewed health providers reckoned that PBF forces them to depart from routine, to be more professional and to respect national norms.^10^ Peer review also helps concerned health staff to improve their practice.

A second causal mechanism identified in the process of this theory-driven evaluation is the fact that PBF appears to be acting on health workers’ motivation through a complex mechanism, all the more since beyond financial premiums, all contractual features and ancillary components of PBF are likely to impact on workers’ various sources of motivation.^21^ Bertone and colleagues^22^ have warned against the risk of looking at PBF payments in isolation, without reference to the overall remuneration of health workers. Their study confirms that the remuneration of health workers is complex and interrelated so that the different financial incentives cannot be examined independently. They have assessed that in Sierra Leone, PBF contributes to about 10% of the total income of health workers – yet, despite this relatively low contribution, workers’ views on the bonuses are positive while views on salary are negative. In Benin, the average monthly salary of clinical health staff at HC level was estimated at about 76 EUR, plus about 11 EUR in premiums in 2011 in the eight districts supported by the World Bank.^23^ No such survey has been recently performed in the districts supported by BTC, but according to the Department of Financial and Material Resources of the Mono-Couffo Departmental Health Directorate, salaries have increased by 25% between 2011 and 2014 throughout civil service. In 2015, the PBF subsidies distributed by BTC to health staff in the three districts in the Mono-Couffo department amounted to 177 415 500 CFA francs (270 468 EUR) – that is, an average of 157 283 CFA francs (240 EUR) per agent, all categories of staff. At the same time, the salary and allowances paid by the State amounted to 990 242 292 CFA francs (1 509 614 EUR), which means that PBF financial premiums represent basically 13% of official staff income (data obtained from the Department of Financial and Material Resources/Departmental Health Office, and BTC “PASS-Sourou” programme). Paul and colleagues^10^ already reckoned that whereas interviewed health staff often referred to financial premiums in their discourse, actually the latter were too weak—and “blurred” into so many others—to have a real, lasting inciting effect. So the authors concluded that PBF motivates health workers through other elements of its “package”: especially, regular formative supervisions enable to strengthen management and clinical capacities, thus play an important role in improving performance; PBF also fosters emulation amongst health facilities as well as improvement in data collection and use of data for management purpose. Moreover, it is important to note that PBF in Benin provides financial incentives to all staff – including support staff – which tends to favour teamwork (eg, motivate cleaners).

### Populations (Health Service Users)

An important aspect of BTC’s HSS programme and interesting feature of its alternative PBF approach is support to organisation, capacity-building, and strong involvement of the demand-side actors through so-called health services users’ platforms. These platforms have been setup and developed in view of defending the rights and strengthening the voice of health services’ users. Their role is not limited to PBF; they also manage complaints from health service users, for instance. After a few years of operation, not only their technical competences, but also their legitimacy, have been strengthened, enabling true dialogue with healthcare providers – which is not the case when contracting out NGOs for punctual tasks.^24^

We have here, the third causal mechanism identified in the process of this theory-driven evaluation: PBF especially appears to be acting through increased accountability due to the strengthened dialogue with the demand-side actors. Indeed, the PBF approach developed was viewed as an opportunity to implement the model of “Local Health System” as described in the Dakar Declaration.^25^ This model updates the health district and implies a multi-actor approach, shared stewardship, as well as a focus on the right to health and increased ownership by the local communities. The BTC alternative PBF approach bestows involvement of health service users’ platforms in PBF governance (through participation in departmental steering committees) and counter-verification, plus other dedicated tasks such as complaint management and follow-up. This has undoubtedly reinforced their legitimacy and contributed to strengthening the interaction and dialogue between supply and demand for healthcare in the districts. Ultimately, this has enabled strengthening of the local health system through more balanced dialogue between service providers and demand side actors, and consequently, it facilitated the search for consensual solutions and problem solving.

### Health Outcomes

**Figure 7:**
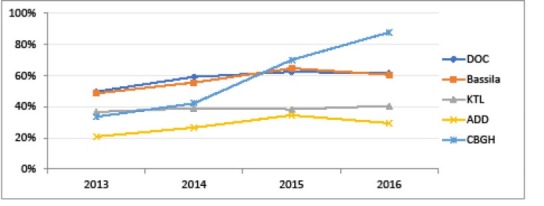
Proportion of Attended Deliveries at HCs Per District, 2013-2016^a^.

Abbreviations: DOC, Djougou-Ouaké-Copargo; KTL, Klouèkanmé-Toviklin-Lalo; ADD, Aplahoué-Djakotomey-Dogbo; CBGH, Comé-Bopa-Grand Popo-Houeyogbé; HCs, health centres.

Source: BTC HSS “PASS-Sourou” programme (PBF validated data).

^a^ 2016 data are extrapolated from the first 3 quarters of the year.

**Figure 8:**
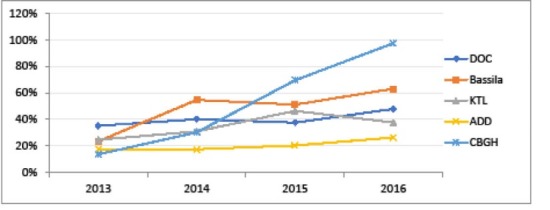
Percentage of Deliveries Referred From HC to DH Per District, 2013-2016^a^.

Abbreviations: DOC, Djougou-Ouaké-Copargo; KTL, Klouèkanmé-Toviklin-Lalo; ADD, Aplahoué-Djakotomey-Dogbo; CBGH, Comé-Bopa-Grand Popo-Houeyogbé; HC, health centre; DH, district hospital.

Source: BTC HSS “PASS-Sourou” programme (PBF validated data).

^a^ 2016 data are extrapolated from the first 3 quarters of the year.

**Figure 9:**
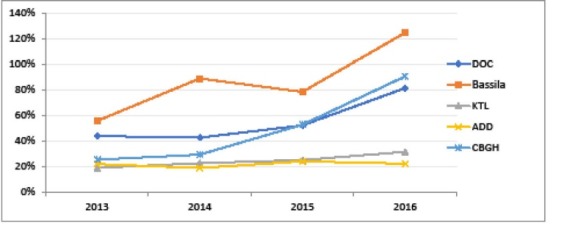
Family Planning Acceptor Rate Per District, 2013-2016^a^.

Abbreviations: DOC, Djougou-Ouaké-Copargo; KTL, Klouèkanmé-Toviklin-Lalo; ADD, Aplahoué-Djakotomey-Dogbo; CBGH, Comé-Bopa-Grand Popo-Houeyogbé.

Source: BTC HSS “PASS-Sourou” programme (PBF validated data).

^a^ 2016 data are extrapolated from the first 3 quarters of the year.

Overall, four years of implementation of PBF in the 5 districts supported by BTC was associated with limited progress in utilisation of health services, but most of all noticeable improvements in some features of quality of care, including user satisfaction. After correcting for autocorrelation, a recent econometric study on panel routine data in Benin shows little and non-significant effects of PBF (considered simplistically as a “yes/no” variable) over a selection of quantitative indicators.^19^ Especially, outpatient attendance is still low in the five districts, ranging from 14.54% to 47.14% in 2015 (coming from resp. 12.78% and 22.28% in 2013) (PBF validated data). Yet, a number of PBF validated indicators followed by BTC (which are more reliable as shown above, but by definition cannot be compared “before and after”) have improved over the three years 2013-2015, and some districts have particularly well improved their performance over the period, as shown in Figures 7-9. Especially, the CBGH district experienced an increase in attended deliveries (resp. deliveries referred from HC to DH; family planning acceptor rate) from 33.49% (resp. 13.67%; 25.52%) in 2013 to 87.65% (resp. 97.57%; 90.53%) in 2016 (data extrapolated from the three first quarters).

**Figure 10:**
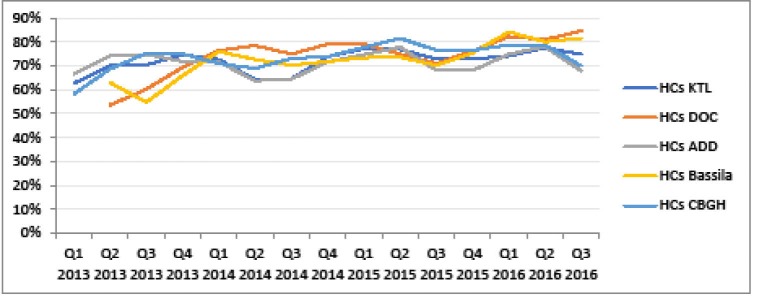
Average Quality Scores at HC Level Per District, Q1 2013-Q3 2016.
Abbreviations: KTL, Klouèkanmé-Toviklin-Lalo; DOC, Djougou-Ouaké-Copargo; ADD, Aplahoué-Djakotomey-Dogbo; CBGH, Comé-Bopa-Grand Popo-Houeyogbé; HCs, health centres.

Source: BTC HSS “PASS-Sourou” programme.

**Figure 11:**
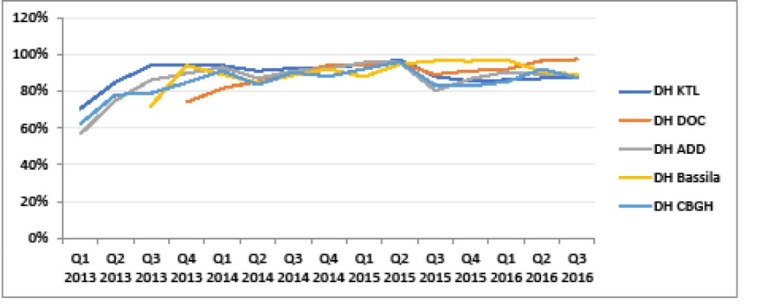
Technical Quality Scores of DHs, Q1 2013-Q3 2016.
Abbreviations: KTL, Klouèkanmé-Toviklin-Lalo; DOC, Djougou-Ouaké-Copargo; ADD, Aplahoué-Djakotomey-Dogbo; CBGH, Comé-Bopa-Grand Popo-Houeyogbé; DHs, district hospitals.

Source: BTC HSS “PASS-Sourou” programme.

**Figure 12:**
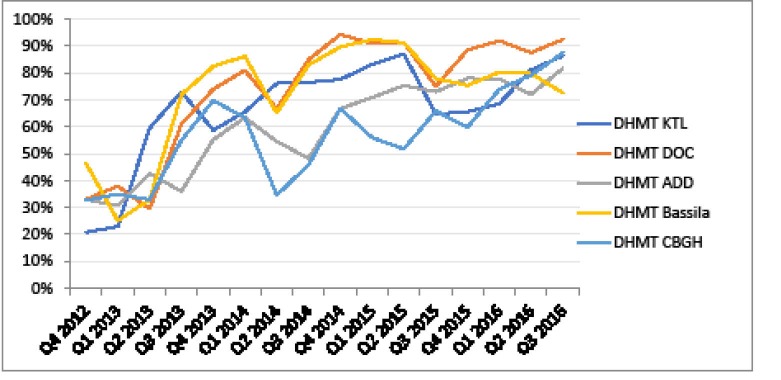
Quality Scores of DHMT, Q4 2012-Q3 2016.
Abbreviations: KTL, Klouèkanmé-Toviklin-Lalo; DOC, Djougou-Ouaké-Copargo; ADD, Aplahoué-Djakotomey-Dogbo; CBGH, Comé-Bopa-Grand Popo-Houeyogbé; DHMT, District Health Management Teams.

Source: BTC HSS “PASS-Sourou” programme.

As for quality improvement, Figures 10-12 show that quality scores have improved at all levels in the five districts. At HC level, unweighted average quality score increased from 65.92% in Q2 2013 to 75.75% in Q3 2016. Unweighted average technical quality score of DHs increased from 63.07% in Q1 2013 (only three hospitals included) to 89.82% in Q3 2016 (all 5 hospitals included). Unweighted average quality score of DHMTs increased from 33.21% in Q4 2012 to 84.18% in Q3 2016.

Mutual trust between populations and healthcare providers has increased as well. User satisfaction rates have already increased on average from 21% in Q3 2015 to 44% in Q1 2016 in the 2 DHs of the Atacora-Donga department, and from 20% in Q3 2015 to 40% in Q1 2016 for the three DHs of the Mono-Couffo department (data collected by the BTC HSS “PASS-Sourou” programme).

## Discussion

### Study Design

Contrary to the World Bank project’s initial design, BTC did not construct its PBF scheme as a randomised control trial, which prevents from demonstrating causal relationships between PBF and observed results. Only “before-and-after” comparisons can be made, and the effect of PBF cannot be isolated since BTC’s project entails various interventions aimed at strengthening the local health system in the five supported districts. Since a quantitative impact assessment does not fit the design of the BTC PBF scheme, we opted for a theory-driven evaluation approach, and identified 3 causal mechanisms through which PBF seem to have had effects and contribute to observed outcomes. Note that other authors found evidence supporting these causal mechanisms elsewhere: some have observed that PBF supported HSS (first mechanism), including leadership, equipment and health management and information system^10,26-28^; an increasing number of studies have pointed the complex effects of PBF over health workers’ motivation in LMICs (second mechanisms)^22,28-32^; and Renmans and colleagues’^5^ framework insist on the fact to consider patients as ‘benefitting principals’ in the PBF contract or scheme (third mechanism relating to dialogue with demand-side actors). Using the Witter and colleagues’ framework^2^ also helped us organise our results in a logical way, enabling to include the context into the analysis. However, other potential ways through which PBF is supposed to impact on results have not been confirmed in Benin, notably increased autonomy at facility level which is still lagging behind – partly because of administrative constraints, but also, as observed in some pilot districts supported by BTC, partly due to reluctance to change and lack of leadership in DHMTs and departmental teams.

### Limitations of the Belgian Development Agency Alternative Belgian Development Agency Approach

The BTC PBF approach is promising and not much resource-consuming in terms of verification: overall, five full time equivalents per year are devoted to verification of results, that is, one full time equivalent per health district covering on average 350 000 inhabitants. However, it also entails some flaws. Firstly, under the current system, the independence of peer verification may be questioned, especially compared to the external verification agency option chosen by the World Bank, Gavi and GFATM. Potential collusion between controllers and controlees is yet balanced by counter-verification at community level (which is not made in the World Bank districts) as well as inclusion of third-parties (notably, mutual health insurance medical officer) in verification teams. Secondly, the difficulty in measuring technical quality of care leads to quite cumbersome matrices and heavy measurement processes. Thirdly, even if BTC intended to build on existing institutions (DHMTs, departmental directorates for health, municipalities, and health service users’ platforms), PBF is not yet totally integrated with other processes. For instance, there are duplications with the many concurrent missions regularly visiting health facilities (in addition to PBF verification and counter-verification, there are monthly DHMIS2 verifications, regular monitoring and supervision missions, etc).

### Cost-Effectiveness

Several authors have warned about the contradiction between the widespread use of PBF and the lack of studies of its costs or cost-effectiveness.^33-35^ A recent study in Tanzania warns against very high incremental costs of PBF (ranging from US$540 to US$907 in the pilot experiment and from US$94 to US$261 for a national programme per additional facility-based birth), as well as to potential substantial opportunity costs diverting attention from service delivery.^28^ The BTC alternative PBF model is not much resource-consuming since we estimated that in 2015, its total cost amounted to 0.59 EUR per person per year. This is far below the usual rule of thumb of an overall output budget of US$3 (2.8 EUR) per person per year recommended in low-income countries.^14^ It has to be noted however that it is complemented by other financial supports by BTC (including other HSS activities), other development partners, and of course the government budget and user fees. Since we have been unable to disentangle PBF and attribute measurable effects to PBF, we cannot estimate the cost-effectiveness of the BTC alternative PBF model. It can just be mentioned that, in the context of the learning process from the joint PBF harmonisation missions, Gavi and the Global Fund mandated an analysis of the PBF effects based on routine data along some selective quantitative indicators. Despite methodological constraints, notably due to poor quality of routine data, it shows no significant differences between the performance of the BTC-supported districts and those under the World Bank for most selected indicators – but even a significantly higher PBF impact on outpatient attendance and antiretroviral therapy initiation for pregnant women in the districts supported by BTC – and this at a much lower cost.^19^

### Sustainability Issues

Despite promising results, PBF sustainability is at risk in Benin, both from institutional and financial points of view. A harmonisation process is under way, but the Beninese authorities have not yet decided on what institutional design PBF should take in the post-donor programme era – which is coming soon, since three development partners’ programme are ending by mid or end of 2017. It is definitely necessary to de-verticalise PBF at the national level (as is intended by the BTC programme), and to integrate it into normal functioning of the health system, as a transversal HSS strategy. However, this will not be an easy task since up to date, PBF is not yet coherent with a number of other reforms. Especially, the relation with the extension of the universal health coverage scheme and quality insurance processes is unclear. The national health financial strategy as well does not clearly state how PBF should position itself vis-à-vis other financing sources (central and deconcentrated government budgets, programme funding, user fees). Moreover, the World Bank approach – which is now implemented in 29 out of 34 districts – is at odds with decentralisation. At the technical level too, integration of PBF requires streamlining its design and reshaping a number of other processes (monitoring, supervisions, etc). As for financial sustainability, the government needs to find sufficient resources – and find appropriate ways of channelling them – to sustain PBF after donors have pulled out.^36^

## Conclusion

This paper shows that an alternative, integrated PBF approach – as compared to the mainstream one promoted by the World Bank PBF Toolkit^14^ – developed with support from BTC in Benin proves to be at the same time less resource-consuming and promising in terms of effects, especially with respect to local HSS (eg, local capacities, infrastructure and equipment), improved technical aspects of healthcare quality, and patient satisfaction. This approach is based on existing institutions that are in charge of governance (at departmental level), verification (through peer review, thus also enabling to mutually strengthen capacities) and counter-verification (through health service users’ platforms that reinforce dialogue between supply and demand in local health systems). It is thus a good basis for both ownership and sustainability. This experience testifies that PBF is not a uniform or rigid model, and opens the policy ground for recipient governments to put their own emphasis and priorities and design ad hoc models adapted to their context specificities.

Future phases of PBF development in Benin will need to take account of the experience accumulated by both interventions, so as to identify the most appropriate, efficient and sustainable design possible. However, several issues still need to be further studied to guide policy-making. In particular, one observes very different patterns of evolution between districts: some of them respond positively to PBF, others stagnate in terms of performance. Hence, the need to better understand the causes of these differences, and take appropriate decisions accordingly. However, integrating PBF within the normal functioning of local health systems, in full coherence with other reforms (decentralisation, human resource development, universal health coverage), remains a big challenge.

## Acknowledgements

This papers stems from collaboration between the BTC-funded PASS-Sourou programme and the “Effi-Santé” research project funded at the University of Liège, Liège, Belgium through the ARC grant for Concerted Research Actions, financed by the French Community of Belgium (Wallonia-Brussels Federation). We would like to thank two reviewers for very useful comments on previous drafts of the paper.

## Ethical issues

The PASS-Sourou programme has been approved by the MoH in Benin as a pilot programme, formally approving to lead research-action activities.

## Competing interests

Authors declare that they have no competing interests.

## Authors’ contributions

All authors contributed to collection and analysis of qualitative data through interviews, participative observation and capitalisation of experiences, as well as to data analysis and elaboration of programme theories. JPK, AEN, MK, and JCA collected primary financial and technical data. EP performed the literature review and drafted the first version of the manuscript. All authors contributed to subsequent and final drafts of the paper and approved its final version.

## Authors’ affiliations

^1^Economie politique et économie de la santé, Faculté des Sciences sociales, Université de Liège, Liège, Belgium. ^2^PASS-Sourou Programme, Belgian Development Agency, Benin. ^3^Comé District, Ministry of Health, Comé, Benin. ^4^Atacora-Donga Departmental Health Team, Ministry of Health, Natitingou, Benin. ^5^Belgian Development Agency, Brussels, Belgium.

## Key messages

**Implications for policy makers**

- Benin has experimented two performance-based financing (PBF) approaches: one under the mainstream World Bank model, and an alternative one which differs substantially in terms of institutions, integration, and associated costs.
- Understanding the mechanisms that explain observed outcomes can help refine the various elements of PBF design according to context specificities.
- It is possible to develop an alternative, less resource consuming PBF approach, grounded on existing domestic institutions and therefore contributing to strengthening local health systems.
- In Benin, PBF enabled to improve healthcare quality but increase in utilisation of health services did not automatically follow suit.
- The alternative PBF approach developed in Benin has helped achieve progress mostly through: comprehensive health system strengthening (HSS) at district level; acting on health workers’ motivation through a complex package of incentives; and increased accountability by reinforcing dialogue with the demand-side actors.

**Implications for public**

A bottom-up performance-based financing (PBF) approach has been developed and implemented in Benin since 2012 as an alternative to the mainstream model supported by several donors. This approach is integrated in existing local institutions (decentralised governance, peer review verification, counter-verification entrusted to health service users’ platforms); it is not much resource consuming and enables to strengthen the local health system. PBF, including its ancillary components, has contributed to strengthen various aspects of the health system (equipment, information system, human resources, governance). After four years of implementation, PBF has led to modest progress in utilisation of health services, but most of all noticeable improvements in quality of care.

## References

[1] Meessen B, Soucat A, Sekabaraga C (2011). Performance-based financing: just a donor fad or a catalyst towards comprehensive health-care reform?. Bull World Health Organ.

[2] Witter S, Toonen J, Meessen B, Kagubare J, Fritsche G, Vaughan K (2013). Performance-based financing as a health system reform: mapping the key dimensions for monitoring and evaluation. BMC Health Serv Res.

[3] Basinga P, Gertler PJ, Binagwaho A (2011). Effect on maternal and child health services in Rwanda of payment to primary health-care providers for performance: an impact evaluation. Lancet.

[4] Rusa L, Ngirabega J, Janssen W, Van Bastelaere S, Porignon D, Vandenbulcke W (2009). Performance-based financing for better quality of services in Rwandan health centres: 3-year experience. Trop Med Int Health.

[5] Renmans D, Holvoet N, Garimoi Orach C, Criel B (2016). Opening the ‘black box’ of performance-based financing in low- and lower middle-income countries: a review of the literature. Health Policy Plan.

[6] Ssengooba F, McPake B, Palmer N (2012). Why performance-based contracting failed in Uganda – an “open-box” evaluation of a complex health system intervention. Soc Sci Med.

[7] Van Belle S, Marchal B, Dubourg D, Kegels G (2010). How to develop a theory-driven evaluation design? Lessons learned from an adolescent sexual and reproductive health programme in West Africa. BMC Public Health.

[8] Belgian Development Agency. Dossier technique et financier – Programme d’appui au secteur de la sante “PASS-Sourou”, Bénin. 2014. Code DGD: NN 3014055. Code navision: BEN 13 025 11.

[9] De Zutter P. Des histoires, des savoirs, des hommes. l’expérience est un capital. Série Dossiers pour un débat n°35. Paris: Eds. Charles-Léopold Meyer; 1994.

[10] Paul E, Sossouhounto N, Eclou DS (2014). Local stakeholders’ perceptions about the introduction of performance-based financing in Benin: a case study in two health districts. Int J Health Policy.

[11] Etats généraux de la santé au Bénin. Rapport général, Cotonou, November 21-24, 2007.

[12] République du Bénin, Ministère de la Santé. Plan national de développement sanitaire 2009-2018.

[13] World Bank. ‘Project Appraisal Document on a Proposed IDA Grant in the Amount of SDR 14.9 million (US$22.8 million equivalent) of which SDR 5.1 million originates from pilot CRW resources (US$7.8 million equivalent) to the Republic of Benin for a Health System Performance Project’, Report No: 53930-BJ; April 9, 2010.

[14] Fritsche GB, Soeters R, Meessen B. Performance-Based Financing Toolkit. Washington, DC: The World Bank; 2014.

[15] République du Bénin, Ministère de la Santé. La pérennisation du financement basé sur les résultats (FBR) dans le secteur de la santé au Bénin. Aide-mémoire de la mission conjointe du 12 au 16 octobre 2015.

[16] République du Bénin, Ministère de la Santé. La pérennisation du financement basé sur les résultats (FBR) dans le secteur de la santé au Bénin. Aide-mémoire de la mission conjointe du 5 au 8 juillet 2016.

[17] Jansen C, Lodi E, Lodenstein E, Toonen J, eds. Vers une couverture maladie universelle au Bénin (Towards universal health coverage in Benin). Amsterdam: KIT Publishers; 2013.

[18] AEDES/Scen-Afrik. PRPSS. Analyse du coût de la vérification et de l’AT dans le cadre du FBR au Bénin; Power Point Presentation, July 2016.

[19] Johnson P, Bello K, Meessen B, Korachais C. Effets des expériences du financement basé sur les résultats (FBR) dans le secteur de la santé au Bénin. Une analyse à partir des données de routine collectées de 2010 à 2015. Institute of Tropical Medicine, Antwerp, and CERRHUD for Gavi and the Global Fund; July 2016.

[20] Sambiéni NE (Dir.). Les mécanismes à l’œuvre dans la production d’effets différenciés du financement basé sur les résultats (FBR) dans le secteur de la santé au Bénin, rapport général (financement: Fonds mondial). Université de Parakou, FLASH; 2016.

[21] Paul E, Renmans D (2017). Performance-based financing in the heath sector in low- and middle-income countries: Is there anything whereof it may be said, see, this is new?. Int J Health Plann Manage.

[22] Bertone MP, M Lagarde, S Witter (2016). Performance-based financing in the context of the complex remuneration of health workers: findings from a mixed-method study in rural Sierra Leone. BMC Health Serv Res.

[23] Lemière C, De Walque D, Ayivi-Guedehoussou N, Juquois M. Evaluation d’Impact du Financement Basé sur les Résultats, Rapport d’analyse de l’enquête de base; 2012.

[24] Ghesquière G, Hounouvi AT, Dramé ML, Gyselinck K, Paul E. Les opportunités du financement basé sur les résultats comme plateforme d’interactions entre l’offre et la demande pour le renforcement du système local de santé au Bénin. Belgian Development Agency (BTC); November 2015.

[25] Community of Practice Health Service Delivery. Renewing health districts for advancing universal health coverage in Africa. Report of the regional conference “Health districts in Africa: progress and perspectives 25 years after the Harare Declaration”; October 21-23, 2013; Dakar, Senegal.

[26] Peerenboom PB, Basenya O, Bossuyt M (2014). La bonne gouvernance dans la réforme du financement du système de santé au Burundi. Santé Publique.

[27] Nahimana E, McBain R, Manzi A (2016). Race to the Top:
evaluation of a novel performance-based financing initiative
to promote healthcare delivery in rural Rwanda. Glob Health
Action.

[28] Bhatnagar A, George AS (2016). Motivating health workers up to a limit: partial effects of performance-based financing on working environments in Nigeria. Health Policy Plan.

[29] Lohmann J, Houlfort N, De Allegri M (2016). Crowding out or no crowding out? A Self-Determination Theory approach to health worker motivation in performance-based financing. Soc Sci Med.

[30] Aninanya GA, Howard N, William JE (2016). Can performance-based incentives improve motivation of nurses and midwives in primary facilities in northern Ghana? A quasi-experimental study. Glob Health Action.

[31] Khim K (2016). Are health workers motivated by income? Job motivation of Cambodian primary health workers implementing performance-based financing. Glob Health Action.

[32] Shen GC, Nguyen HTH, Das A, Sachingongu N, Chansa C, Qamruddin J, Friedman J (2017). Incentives to change: effects of performance-based financing on health workers in Zambia. Hum Resour Health.

[33] Kalk A, Paul FA, Grabosch E (2010). ‘Paying for performance’ in Rwanda: does it pay off?. Trop Med Int Health.

[34] Ireland M, Paul E, Dujardin B (2011). Can performance-based financing be used to reform health systems in developing countries?. Bull World Health Organ.

[35] Borghi J, Little R, Binyaruka P, Patouillard E, Kuwawenaruwa A (2015). In Tanzania, the many costs of pay-for-performance leave open to debate whether the strategy is cost-effective. Health Affairs.

[36] Paul E. Marché de services relatif à réalisation d’une étude sur la viabilité et la pérennisation de l’approche du Financement Basé sur les Résultats (FBR) au Bénin – Rapport final, CSC BTC/CTB BEN 405. Published July 18, 2016.

